# A systematic review of assisted and third-party reproduction guidelines regarding management and care of donors

**DOI:** 10.1186/s12978-024-01804-2

**Published:** 2024-06-01

**Authors:** Elnaz Iranifard, Samira Ebrahimzadeh Zagami, Malihe Amirian, Hossein Ebrahimipour, Robab Latifnejad Roudsari

**Affiliations:** 1 Student Research Committee, Mashhad University of Medical Sciences, 9137913199 Mashhad, Iran; 2 Nursing and Midwifery Care Research Center, Mashad University of Medical Sciences, 9177949025 Mashhad, Iran; 3 Department of Midwifery, School of Nursing and Midwifery, Mashhad University of Medical Sciences, 9137913199 Iran Mashhad,; 4 Department of Obstetrics and Gynecology, Fellowship of Infertility, School of Medicine, Milad Infertility Treatment Center of Mashhad, University of Medical Sciences, Mashhad, 9137913316 Iran; 5 Department of Health Economics and Management, School of Health, Mashhad University of Medical Sciences, 9196773113 Mashhad, Iran; 6 Health Sciences Research Center, Torbat Heydarieh University of Medical Sciences, 9519633787 Torbat heydarieh, Iran

**Keywords:** Egg donor, Embryo donor, Gamete donor, Guidelines, Reproductive donation, Sperm donor, Third-party reproduction

## Abstract

**Background:**

Gamete and embryo donors face complex challenges affecting their health and quality of life. Healthcare providers need access to well-structured, evidence-based, and needs-based guidance to care for gamete and embryo donors. Therefore, this systematic review aimed to synthesize current assisted and third-party reproduction guidelines regarding management and care of donors.

**Methods:**

The databases of ISI, PubMed, Scopus, and websites of organizations related to the assisted reproduction were searched using the keywords of “third party reproduction”, “gamete donation”, “embryo donation”, “guidelines”, “committee opinion”, and “best practice”, without time limit up to July 2023. All the clinical or ethical guidelines and best practice statements regarding management and care for gamete and embryo donors written in the English language were included in the study. Quality assessment was carried using AGREE II tool. Included documents were reviewed and extracted data were narratively synthesized.

**Results:**

In this systematic review 14 related documents were reviewed of which eight were guidelines, three were practice codes and three were committee opinions. Five documents were developed in the United States, three in Canada, two in the United Kingdom, one in Australia, and one in Australia and New Zealand. Also, two guidelines developed by the European Society of Human Reproduction and Embryology were found. Management and care provided for donors were classified into four categories including screening, counseling, information provision, and ethical considerations.

**Conclusion:**

While the current guidelines include some recommendations regarding the management and care of gamete/embryo donors in screening, counseling, information provision, and ethical considerations, nevertheless some shortcomings need to be addressed including donors’ psychosocial needs, long-term effects of donation, donors’ follow-up cares, and legal and human rights aspects of donation. Therefore, it is needed to conduct robust and well-designed research studies to fill the knowledge gap about gamete and embryo donors’ needs, to inform current practices by developing evidence-based guidelines.

**Supplementary Information:**

The online version contains supplementary material available at 10.1186/s12978-024-01804-2.

## Abstract (plain language summary)

Gamete and embryo donors face complex challenges affecting their health and quality of life. To manage these challenges, healthcare providers need guidelines that are based on evidence and donors’ real needs. In order to develop a comprehensive guideline that meets the needs of donors; it is important to review the current guidelines. So, in this study we reviewed the current assisted and third-party reproduction guidelines regarding management and care of donors. We searched databases and relevant websites and found 14 related documents. The main topics recommended for management and care of donors in these guidelines included screening, counseling, information provision, and ethical considerations. We recognized that some of donors’ needs are neglected in these documents including donors’ psychosocial needs, long-term effects of donation on donors, their follow-up cares, and legal and human rights aspects of donation. Therefore, there is need for further research to develop guidelines based on donors’ unmet needs.

**Supplementary Information:**

The online version contains supplementary material available at 10.1186/s12978-024-01804-2.

## Introduction

Reproductive donation is defined as using an egg, sperm, or embryo that have been donated by a third person (donor) in order to conceive a child in a person or couple who are not able to reproduce themselves [1–3]. The use of reproductive donation has led to thousands of birth worldwide [4, 5].

Reproductive donation is faced with various challenges in different aspects, such as recruitment and screening of donors, gaining informed consent from involved parties, conflict of interest among involved parties, sociocultural problems as well as religious, ethical, and legal concerns [6–12]. Gamete or embryo donation can also have adverse effects on the health and well-being of donors. Physical side effects related to the ovarian stimulation, psychological stress as a result of feeling responsibility and attachment to the donor-convinced child, fear of the revelation of identity, regretting the donation decision, and social burdens like stigma related to the gamete and sperm donation are among some of the psycho-social concerns of third-party reproduction, that could affect the health and well-being of donors [6, 7, 9, 13–17]. Therefore, it is important to understand the needs of gamete and embryo donors to prepare them for the donation process and its possible side effects.

Over the past decades, guidelines have become a valuable tool for the synthesis of health and care-related information [18, 19]. Guidelines are a summary of current medical, psychosocial and ethical information as well as knowledge in the form of structured and evidence-based recommendations for care providers and patients/clients about specific circumstances, such as diagnosis, treatment, or care. Guidelines need to be revised regularly to meet the constant development of evidence [18–23]. Although guidelines are not usually legally binding, deviations from them must be justified [20]. World Health Organization describes guidelines as recommendations intended to assist recipients and providers of health care and other parties involved to make informed decisions; by providing information about what should be done by each party involved [24].

Gamete and embryo donors should be considered as patients/clients by the fertility clinics [25]. Only when the programs see donors as patients/clients the needs and experiences of donors becomes a necessary component of care [25, 26]. Gamete and embryo donors go through medical interventions such as physical or psychological screenings or blood tests, that can be challenging for them; also donors may experience adverse effects of donation process on their physical, mental or even social health [5, 25, 27–29]. Based on the challenges of third-party reproduction and the possible adverse effects they can have on the health, well-being, and quality of life of donors [5, 25, 27–29], it is important for healthcare providers to have access to well-structured, evidence-based guidelines to care for gamete and embryo donors’ [4, 26].

Although respected organizations in the field of infertility treatment such as American Society for Reproductive Medicine (ASRM), European Society of Human Reproduction and Embryology (ESHRE), and Human Fertilization and Embryology Authority (HFEA) have published guidelines regarding the third party reproduction including care provided for donors [30–34]; various studies from different countries have reported that gamete and embryo donors’ needs and desires have not been met. These studies suggest that at least some donors are receiving insufficient information about practical issues and future consequences of their donation; also, they do not receive proper counseling as needed before, during and/or after donation, or receive no/limited support [12, 25, 26, 35, 36].

As mentioned already, evidence suggests that gamete and embryo donors’ needs are not being fully met, and clinical practice regarding donors must be improved. Also the lack of a comprehensive, donor-centered guideline which focuses solely on the gamete and embryo donors’ needs is evident, especially in developing countries, where donors are more likely to be of lower socioeconomic status with limited knowledge and information about the donation process [4, 37]. Therefore, to allow a better understanding of current practice, this systematic review was conducted to provide a synthesis of the current assisted and third-party reproduction guidelines regarding management and care of donors.

## Methods

This systematic review was conducted based on the Preferred Reporting Items for Systematic Reviews and Meta-analysis (PRISMA) [38]. The protocol of this systematic review is registered in PROSPERO (international prospective register of systematic reviews) under the code of CRD42023474241.

### Search strategy and data sources

The electronic databases of Science Citation Index, PubMed, and Scopus were searched by two researchers (EI, MA), independently, using search strings that included keywords/MESH terms of “third party reproduction”, “gamete donation”, “embryo donation”, “guidelines”, “committee opinion”, and “best practice” as well as Boolean operators of AND/OR, and punctuation tricks (quotation marks), without time limit up to July 2023. Search strategy of electronic databases is available in Additional file 1.

Websites that publish guidelines including Guidelines International Network (www.g-i-n.net), National Institute for Health and Clinical Excellence (www.nice.org.uk), and National Guidelines Clearinghouse (www.guideline.gov); and organizations related to the assisted reproduction techniques including Human Fertilization and Embryology Authority (HFEA), American Society for Reproductive Medicine (ASRM), European Society of Human Reproduction and Embryology (ESHRE), Canadian Fertility and Andrology Society (CFAS), and Fertility society of Australia and New Zealand were manually searched. It should be noted that the organizations related to assisted reproductive techniques were selected based on the appearance of their names in the study selection phase of the initial database search.

After removing the duplicate records, the remaining documents were assessed for inclusion criteria by two authors (EI, MA), independently. All the clinical or ethical guidelines and best practice statements regarding management and care of gamete and embryo donors, written in the English language were included in the study. Guidelines and best practice statements that partly dealt with the subject of management and care of gamete and embryo donors were also included. If there were more than one revision of guidelines, only the latest version was included. Documents other than clinical or ethical guidelines, best practice recommendations and or committee opinions, documents regarding other parties in third-party reproduction (e.g., recipients, healthcare providers, and/or children conceived by third-party reproduction), documents in regards with other aspects of assisted reproduction, documents regarding gamete and embryo donation for purposes other than reproduction (e.g., research) and documents in languages other than English were excluded. A senior researcher (RLR) supervised the data selection process.

### Quality assessment

Quality assessment is an important step in writing a systematic review [39, 40]. In this systematic review the quality of included documents was assessed by two researchers independently (EI, SEZ), using Appraisal of Guidelines for Research and Evaluation II (AGREE II) instrument, which is designed to assess the quality of guidelines, provide direction on guideline development, and guide what specific information ought to be reported in the guidelines [40, 41]. AGREE II is a 23-item tool comprising six quality-related domains including scope and purpose, stakeholder involvement, rigor of development, clarity of presentation, applicability, and editorial independence; followed by two global rating items. Each item is rated on a 7-point scale. After quality appraisal of each document, based on the 23-item tool, researchers (EI, SEZ, RLR) calculated the quality score for each of the six domains based on the instrument user manual [41]. Finally, a score (1 to 7, with 1 lowest and 7 highest possible overall score) for overall quality of the guidelines were assigned by the research team based on the scores of all six domains. The overall quality of the documents were also categorized based on the overall score and research team agreement as extremely good (7): if all domains scored ≥ 75%; very good (6): if more than 3 domains scored ≥ 75% and other domains scored ≥ 50%; good (5) if at least one domain scored ≥ 75% and others scored ≥ 25%; moderate (4): if more than 3 domains scored ≥ 50% and others scored ≥ 25%; poor (3): if at least 3 domains socred ≥ 50%; very poor (2): if at least 3 domains scored > 25%, and extremely poor (1): if all domains scored ≤ 25. The quality assessment score of each document and AGREE II Score Sheet can be accessed through Additional file 2 and 3, respectively.

### Data extraction and analysis

Full texts of documents that met the inclusion criteria were retrieved and reviewed. Data were extracted and tabulated based on the pre-prepared self-structured checklist, including the name of the guideline, publishing organization, year of publication, country of publication, and clinical and/or ethical recommendations regarding management and care of gamete and embryo donors. The data extraction process was carried on by two researchers (EI, HE) working together. In case of any disagreement between the two researchers, a senior researcher (RLR) commented on the extracted data.

### Data analysis

*‘*Narrative synthesis’ was used to analyse the data in this study, which refers to an approach to the systematic review and synthesis of findings from multiple studies that primarily use of words and text to summarize the findings of the synthesis. Indeed, its main characteristic is adopting a textual approach to the process of synthesis to ‘tell the story’ of the findings. It can focus on a wide range of questions, and not only those relating to the effectiveness of a particular intervention [42]. For this purpose, the included guidelines and good practice statements were summarized and synthesized narratively by three researchers working together (EI, SEZ, RLR). As mentioned, data were extracted regarding management/care provided for gamete and embryo donors into spreadsheets. Similar recommendations from different guidelines were put into same columns, and were given a label, then those labels were compared with each other and the labels which point to similar type of management and care were merged into categories. Finaly, recommended clinical and/or ethical management and care of gamete and embryo donors were categorized into four main categories which will be discussed in the ‘result’ section.

## Results

### Search results

**Fig. 1:**
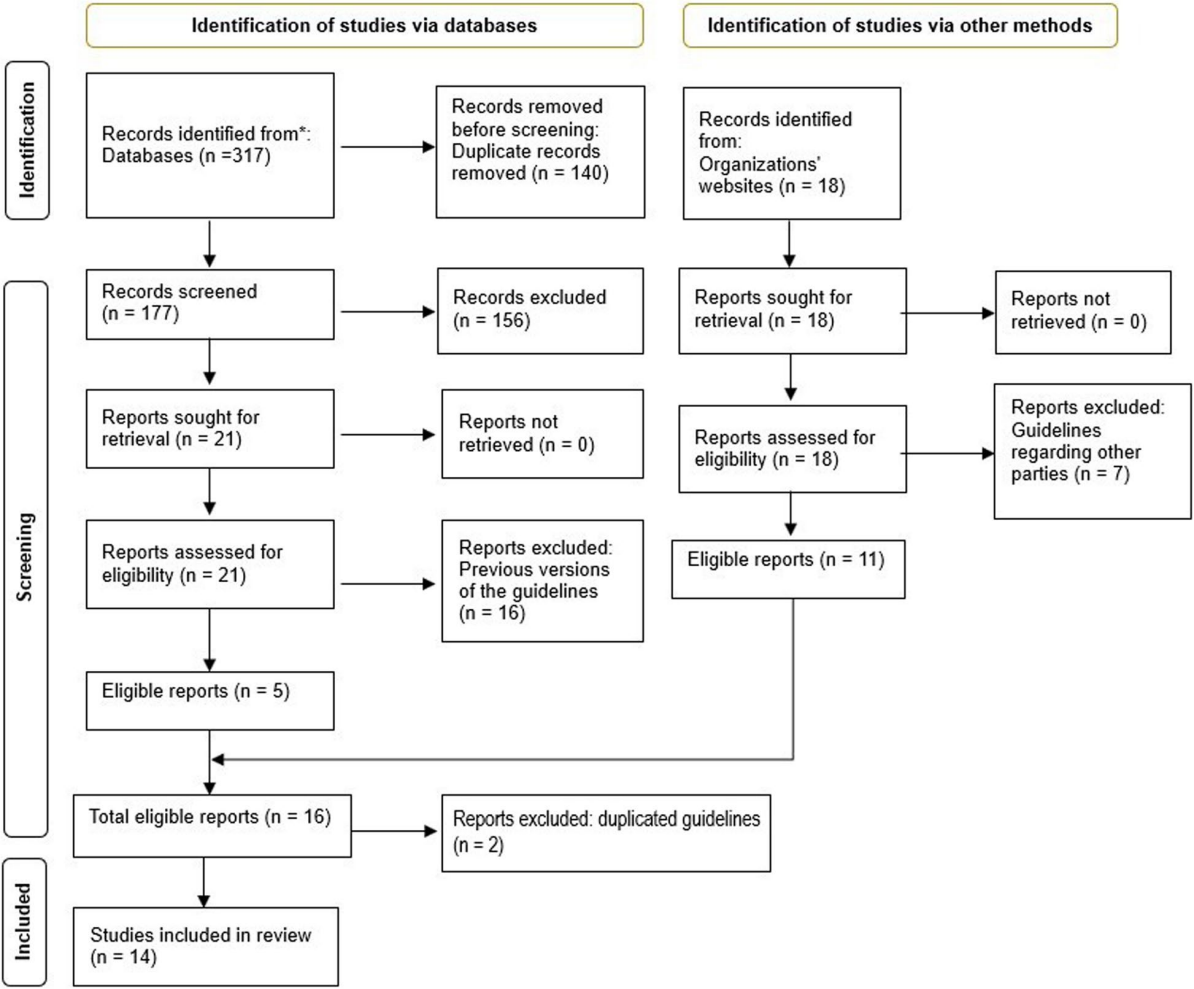
PRISMA 2020 flowchart of study selection

Three hundred seventeen studies were identified through electronic databases (ISI: 104, Scopus: 154, and PubMed: 59). After removing the duplicate studies, the title and abstracts of 177 articles were reviewed, from which 156 articles did not meet the inclusion criteria, and 21 articles were assessed for eligibility. 16 articles were previous versions of included guidelines. Eventually, 5 studies that met the inclusion criteria were retrieved. In addition, 11 guidelines were retrieved through related organizational websites, and after the removal of duplicate guidelines (*n* = 2), the remaining 14 guidelines that met the inclusion criteria, were included in the review (Fig. 1).

### Guidelines characteristics

Fourteen documents were included in this review of which eight were guidelines [30, 43–49], three were practice codes [34, 50, 51], and three were committee opinions [52–54]. Five guidelines were developed in the United States [30, 44, 52–54], three Canada [45–47], two in the United Kingdom [34, 48], and one in Australia [49] and one guideline in Australia and New Zealand [50] were identified. Also, two guidelines developed by the European Society of Human Reproduction and Embryology (26, 36) were identified. The majority of guidelines (64%) were developed in the last five years (*n* = 9). Guidelines were also categorized by the research team based on their focus on the gamete and embryo donors. If a guideline specifically was developed regarding gamete/embryo donors, it was considered as totally focused; if the document was about third-party reproduction and included some content related to the recipients and/or donation offspring, it was considered partly focused; and if the guidelines were about infertility treatment in general with some content on donors, it was considered slightly focused. The characteristics of the guidelines are available in Table 1.

**Table 1:** Characteristics of clinical and ethical guidelines regarding gamete and embryo donors

*	Name	Organization	Year	Country/ Region	Focus on content	AGREE-II overall score
1	Guidelines for counselling in infertility [43]	ESHRE^a^	2001	Europe	Slightly	3 (poor)
2	Psychological guidelines for embryo donation [44]	ASRM^b^	2002	USA	Partly	2 (very poor)
3	Assisted Human Reproduction Counselling Practice Guidelines [45]	CFAS^c^	2009	Canada	Slightly	2 (very poor)
4	Guidelines for the Donation of Gametes and Embryos, Surrogacy and Preimplantation Genetic Diagnosis [46]	CEST^d^	2009	Canada (Quebec)	Partly	3 (poor)
5	Guidelines for Third Party Reproduction [47]*	CFAS	2016	Canada	Partly	5 (good)
6	Interests, obligations, and rights in gamete and embryo donation: an Ethics Committee opinion [52]	ASRM	2019	USA	Partly	3 (poor)
7	UK guidelines for the medical and laboratory procurement and use of sperm, oocyte and embryo donors [48]	ACE^e^, ABA^f^, BFS^g^, BAS^h^	2019	UK	Totally	3 (poor)
8	Repetitive oocyte donation: a committee opinion [53]	ASRM, SART^i^	2020	USA	Totally	3 (poor)
9	Financial compensation of oocyte donors: an Ethics Committee opinion [54]	ASRM	2021	USA	Totally	3 (poor)
10	Guidance regarding gamete and embryo donation [30]	ASRM, SART	2021	USA	Partly	6 (very good)
11	Code of practice (9th edition) [34]	HFEA^j^	2021	UK	Slightly	5 (good)
12	Code of practice for assisted reproductive technology units [50]	RTAC^k^	2021	Australia and New-Zealand	Slightly	5 (good)
13	Good practice recommendations for information provision for those involved in reproductive donation [51]	ESHRH	2022	Europe	Partly	6 (very good)
14	Ethical guidelines on the use of assisted reproductive technology [49]	NHMRC^l^	2023	Australia	Slightly	5 (good)

^a^European Society of Human Reproduction and Embryology, ^b^American Society for Reproductive Medicine, ^c^Canadian Fertility and Andrology Society, ^d^Commission de l’éthique de la science et de la technologie, ^e^Association of Clinical Embryologists, ^f^Association of Biomedical Andrologists, ^g^British Fertility Society, ^h^British Andrology Society, ^i^Society for Assisted Reproductive Technology, ^j^Human Fertilisation and Embryology Authority, ^k^Reproductive Technology Accreditation Committee, ^l^National Health and Medical Research Council

^*^Please note that this guideline is currently under revision

Two guidelines were considered of very good quality with overall AGREE II score of 6. Four guidelines were of good quality (overall AGREE II score = 5). While six documents were of poor quality (overall AGREE II score = 3). Also, two guidelines were considered very poor in quality (overall AGREE II score = 2). It must be noted that among the poor-quality documents, there were three committee opinions, which can justify their low score, since some items of the AGREE II tool did not apply to these types of documents. Quality assessment score of each domain and overall score can be found in Additional File 2.

### Main findings

Four main categories of management and care provided for gamete and embryo donors were identified including (1) screening (2) counseling (3) information provision and (4) ethical considerations. These categories will be further discussed.

### Screening

According to the reviewed guidelines gamete and embryo donors must be screened before donation to ensure the safety and well-being of all parties involved in third-party reproduction. Screening guidelines provide evidence-based eligibility and exclusion criteria for potential donors. Although there are variations among guidelines, the screening process mainly consists of taking medical history, physical exams, infectious diseases screening, genetic screening, and psychosocial screening (Table 2).

Guidelines recommend taking potential donors’ medical history including surgical history and if relevant, the medical history of their family. In such a way, not only ineligible people are excluded but also health-care providers can assess possible risks due to the donation that can influence potential donors’ health based on their medical history [30, 34, 46–48].

Physical examination of potential donors including pelvic examination of oocyte donors is also recommended by included guidelines [30, 34, 47, 48].

Based on the reviewed guidelines to minimize the risk of infection transmission among gamete/embryo donors, recipients, and donation offspring; it is important to screen potential donors for infectious diseases [30, 34, 47–50]. Four guidelines provide detailed recommendations regarding infectious disease, including screening for infectious disease, treatment, re-screening and quarantine period needed for provided gametes and/or embryos before using them in donation [30, 34, 47, 48]. Tests of HIV-1 and HIV-2 antibody, Hepatitis B antigen and antibody (IgG, IgM), Hepatitis C antibody, and serology for syphilis, except of one guideline that recommends it only in sperm donors [46] is recommended for all potential gamete/embryo donors [30, 34, 46–48]. While two guidelines recommend routine screening for chlamydia and gonorrhea in all donors [30, 48], CEST’s guideline recommends routine chlamydia and gonorrhea screening only in sperm donors [46], HFEA’s ‘Code of Practice’ advises just routine chlamydia screening only in sperm donors [34], and CFAS’s ‘Guideline on Third-party Reproduction’ recommends routine gonorrhea screening for only female donors [47]. Screening for HTLV types I and II, and cytomegalovirus (CMV) (IgG, IgM) are more controversial. CEST’s guidelines recommend routine HTLV and CMV screening only in sperm donors [46]. CFAS’s ‘Guideline on Third-party Reproduction’ recommends CMV screening in all male donors and HTLV screening in male embryo donors [47]. ASRM’s Guidance regarding gamete and embryo donation recommends CMV and HTLV screening in all male donors [30]. HFEA’s ‘Code of Practice’ advises screening for CMV based on the medical history of the donor and HTLV screening based on both medical history and birth or residing country of donors [34]. Just one guideline recommends routine CMV and HTLV screening in all donors [48].

Some documents recommended additional testing. Both British guidelines recommend further evaluation for infectious diseases such as HPV and HSV based on medical history, if needed [34, 48]. Canadian guidelines recommend ABO and Rh screening in all donors, trachomatis in female donors, and ovarian reserve tests in oocyte donors [47].

Five guidelines recommend completing a comprehensive genetic/heredity disease questionnaire for potential donors and screening them for genetic diseases to exclude potential donors with genetic/chromosomal defects [30, 34, 46–48].

It is recommended by some of the included guidelines that fertility clinics establish a mechanism to update and monitor the health status of the gamete/embryo donors including medical and genetic disease history [30, 52].

According to the included guidelines psychosocial and mental health screening of potential donors is another important part of the donation process, and using validated questionnaires and/or tests and interviewing potential donors in order to identify any absolute or relative exclusion criteria is seen as crucial [30, 34, 43–48].

**Table 2:** Screening tests recommended by different guidelines for the potential gamete/embryo donors

*	Name	Medical history	Physical exam	Infectious disease screening								Genetic screening	Psychosocial screening	Additional tests
				HIV	HBV	HCV	Syphilis	HTLV	CMV	Gonorrhea	Chlamydia			
1	Guidelines for counselling in infertility [43]	-	-	-	-	-	-	-	-	-	-	-	+	-
2	Psychological guidelines for embryo donation [44]	-	-	-	-	-	-	-	-	-	-	-	+	-
3	Assisted Human Reproduction Counselling Practice Guidelines [45]	-	-	-	-	-	-	-	-	-	-	-	+	-
4	Guidelines for the Donation of Gametes and Embryos, Surrogacy and Preimplantation Genetic Diagnosis [46]	+	-	All donors	All donors	All donors	Sperm donor	Sperm donor	Sperm donor	Sperm donor	Sperm donor	+	+	Blood test
5	Guidelines for Third Party Reproduction [47]	+	+	All donors	All donors	All donors	All donors	Male embryo donor	Male donor	Female donor	-	+	+	ABO Rh, Trachomatis female donors, Ovarian reserve tests
6	UK guidelines for the medical and laboratory procurement and use of sperm, oocyte and embryo donors [48]	+	+	All donors	All donors	All donors	All donors	All donors	All donors	All donors	All donors	+	-	Additional viral infections test
7	Guidance regarding gamete and embryo donation [30]	+	+	All donors	All donors	All donors	All donors	Male donor	Male donor	+	+	+	+	-
8	Code of practice (9th edition) [34]	+	+	All donors	All donors	All donors	All donors	Based on history	Based on history	-	Sperm donor	+	+	Additional infectious disease test

(‘+’ means that there are recommendations in the guideline about this topic, ‘- ‘means that this topic has not been mentioned in the guideline)

### Counseling

According to some of the included guidelines the decision to donate gamete/embryo is complicated and donors would benefit from psychological counseling [30, 34, 51]. In earlier guidelines, counseling was carried out to screen potential donors’ mental/psychological health, but the latest guidelines have separated counseling from psychological screening. These guidelines recommend that counseling should be separated from mental screening and/or information provision [34, 48, 49, 51]. Counseling for donors consists of donation motivation, donation implications on donors’ life, contact with donation offspring, legal issues, time of counseling, providing support, and special concerns (Table 3).

Approaches to counseling vary among countries [51]. As per included guidelines counseling is mandatory for all parties involved in third-party reproduction in Australia, Canada, New Zealand, and the United Kingdom [34, 47, 49, 50]; although counseling is not mandatory in the United States, ASRM strongly recommends it [30].

It has been highlighted in the majority of guidelines that during counseling sessions, the counselor must discuss donors’ intentions and motivations to make sure that donors are not coerced or under pressure to donate [30, 43–45, 47, 49]. Included guidelines emphasis that counselors also should discuss donation implications on donors’ lives, their partners, and their current and/or future children [30, 34, 43, 45, 49, 51]. Also, it has been emphasized in most of the guidelines that it is important to talk through anonymous donation and its consequences. If anonymity is not optional or the donor is open to information sharing, donors’ expectations, needs, and preferences regarding possible future contact with donation offspring must be discussed [30, 34, 43, 45, 49, 51, 52].

Regarding counseling on legal issues, it has been recommended by included guidelines that counseling on legal issues including donors’ legal rights and responsibilities or donor-conceived child legal parents, must be provided for donors and they must be encouraged to get such counseling [30, 34, 43–45, 47, 49, 51].

Four guidelines emphasized that counseling must be provided before, during, and after donation [34, 43, 45, 51]. However, HFEA’s code of practice recommends that counseling should be provided at any time on donors’ request [34]. Good practice recommendations for information provision for those involved in reproductive donation, by ESHRE, recommends counseling should be also provided before, during, and after contact with the donation offspring to donors and their family members [51]. CFAS recommends follow-up care for oocyte donors, but does not provide more details about it [47].

Based on the included guidelines among donors, there are groups with special concerns, and their special needs must be met during counseling sessions provided by fertility centers. For instance, known donors must receive counseling both in sessions without the presences of the recipients and joined sessions with the recipients. They must also be counseled about their feelings toward the donation offspring, the effect of treatment failure on the donor, the donors’ role in donation offspring life, family dynamics, and setting boundaries with recipient families [30, 43, 45, 51]. Embryo donors have also special needs. The donor couple must be counseled in joined and private sessions, with emphasis on the biological relationship between the donor couple and the donation offspring. They should also receive counseling regarding the implications of having a full biological sibling on their own children [43, 45]. Based on ESHRE’s ‘good practice recommendations for information provision for those involved in reproductive donation’, Egg-share donors and embryo donors who are under infertility treatment themselves must be counseled about the impact of a possible failed ART while recipients have a successful treatment with the oocyte or embryo they provided [51].

Two guidelines have pointed out that it is also important to provide support for donors, which could be in the form of support groups and must be culturally and religiously sensitive [34, 49]. Regarding donors’ family, two guidelines namely HFEA’s ‘code of practice’ and ESHRE’s ‘good practice recommendations for information provision for those involved in reproductive donation’, have suggested that if requested, donors’ families must be provided with counseling too [34, 51].

**Table 3:** Content of counseling recommended by different guidelines for gamete/embryo donors

*	Name	Donation motivation	Donation implications	Contact with donation offspring	Legal issues	Time of counseling	Special concerns
1	Guidelines for counselling in infertility [43]	+	+	+	+	Before, during, and after donation	Known donors, embryo donors
2	Psychological guidelines for embryo donation [44]	+	-	-	+	-	-
3	Assisted Human Reproduction Counselling Practice Guidelines [45]	+	+	+	+	Before, during, and after donation	Known donors, embryo donors
4	Guidelines for Third Party Reproduction [47]	+	-	-	+	Follow-up care	-
5	Guidance regarding gamete and embryo donation [30]	+	+	+	+	-	Known donors
6	Code of practice (9th edition) [34]	-	+	+	+	Before, during, and after donation	-
7	Good practice recommendations for information provision for those involved in reproductive donation [51]	-	+	+	+	Before, during, and after donation	Known donors, Egg share donors
8	Ethical guidelines on the use of assisted reproductive technology [49]	+	+	+	+	-	-

(‘+’ means that there are recommendations in the guideline about this topic, ‘- ‘means that this topic has not been mentioned in the guideline)

### Information provision

Guidelines have recommended that in order to make an informed decision all donors must access up-to-date, cultural, and religious sensitive information in an appropriate and understandable language [34, 49–51]. Donors must also receive information about the donation procedures, side effects, screening results, and responsibilities and rights (Table 4).

Also it is emphasized by included guidelines that fertility centers must provide information about donation procedures to donors, including examination and screening tests and the reason those are done, instructions for medication usage and/or necessary lifestyle modifications, and medical procedures that will take place [30, 34, 43–47, 50, 51]. These guidelines have also highlighted that fertility centers are obligated to inform potential donors of all possible side effects related to gamete/embryo donation on the physical, reproductive, mental, and/or social health of donors including the possibility of ovarian hyper-stimulation syndrome and pregnancy (if donor is sexually active) in oocyte donors; also it must be disclosed to potential donors that due to lack of evidence, long-term side effects of donation are not fully known yet [30, 34, 43–47, 50–52].

As mentioned before potential donors must go through the screening process, including infectious disease, mental health, and genetic screenings; included guidelines recommend fertility centers must inform the potential donors of the results of these screening tests, and if necessary the fertility centers must also provide consulting, treatment, and referrals for potential donors [30, 34, 47, 48, 50–52].

Most of the guidelines have pointed out that donors must be given information regarding their responsibilities and rights, including commitment to the process, commitment to update their health status that can affect donor offspring, or provide and update their contact information when donating in a system of identifiable/non-anonymous donation [30, 34, 45, 46, 49–52]. Three guidelines, ‘Guidance regarding gamete and embryo donation’, ‘Guidelines for Third Party Reproduction’, and ‘Good practice recommendations for information provision for those involved in reproductive donation’, have suggested that donors must be informed about the possibility of donor offspring [OR THEIR PARENTS] accessing them [OR THEIR FAMILY MEMBERS, AT ANY TIME] through direct-to-consumer DNA tests; even when the donation has been carried out anonymously [30, 47, 51].

**Table 4:** Information provision recommended by different guidelines for gamete/embryo donors

*	Name	Procedure	Side effects	Screening results	Responsibilities and rights
1	Guidelines for counselling in infertility [43]	+	+	-	-
2	Psychological guidelines for embryo donation [44]	+	+	-	-
3	Assisted Human Reproduction Counselling Practice Guidelines [45]	+	+	-	+
4	Guidelines for the Donation of Gametes and Embryos, Surrogacy and Preimplantation Genetic Diagnosis [46]	+	+	-	+
5	Guidelines for Third Party Reproduction [47]	+	+	+	+
6	Interests, obligations, and rights in gamete and embryo donation: an Ethics Committee opinion [52]	-	+	+	-
7	UK guidelines for the medical and laboratory procurement and use of sperm, oocyte and embryo donors [48]	-	-	+	-
8	Guidance regarding gamete and embryo donation [30]	+	+	+	+
9	Code of practice (9th edition) [34]	+	+	+	+
10	Code of practice for assisted reproductive technology units [50]	+	+	+	+
11	Good practice recommendations for information provision for those involved in reproductive donation [51]	+	+	+	+
12	Ethical guidelines on the use of assisted reproductive technology [49]	-	+	-	+

(‘+’ means that there are recommendations in the guideline about this topic, ‘- ‘means that this topic has not been mentioned in the guideline)

### Ethical considerations

The reviewed guidelines offered recommendations on ethical aspects of management and care of gamete and/or embryo donors to ensure respect for the donors’ well-being, dignity, and human rights in third-party reproduction. These ethical aspects include recommendations on obtaining informed consent, donors’ age limit, repetitive donation restriction, donors’ right to know the donation outcome, conditional donation, and compensation (Table 5).

The majority of guidelines recommended that fertility centers must obtain informed consent from donors for all the procedures done during gamete/embryo donation including screening tests, egg retrieval, and sharing information with recipients, and they must make sure that decision to donate gamete/embryo was made by free will, and not under coercion or pressure [30, 34, 43–52]. Four guidelines addressed donors’ partners consent and pointed out that while there is no need to obtain consent from gamete donors’ partners, donors must be encouraged to talk about their decision to donate with their partners [34, 43, 45, 49]. Based on guidelines, there should be enough time between each step of information provision, obtaining consent, and initiating donation to ensure donors informed decision-making [34, 44, 49]. Donors must also be informed about how and at which stages of donation they can withdraw their consent [34, 45, 47, 49, 51, 52].

Some guidelines recommend minimum and maximum age limits for donors, the former is set to confirm donors’ ability to give informed consent and the latter is set to ensure the quality of provided gametes [30, 34, 44, 46–49]. American guidelines set the lower age limit to 21 years, they also recommend informing the recipient couple if the donor is of older age e.g. oocyte donor older than 34 [30, 44]. For British donors, 18 to 35 for egg donors and 18 to 45 for sperm donors is the age limit [34, 48]. Canadian guidelines recommend a lower age limit of 18 for all donors; and upper age limit of 35 for egg donors and 40 for sperm donors [46, 47]. Australian guidelines set the lower age limit to 18 years, they also recommend informing the recipient couple if the donor is of older age [49].

Most of the guidelines have emphasized that for the safety of (oocyte) donors and to avoid the risk of inadvertent consanguineous relationships, repetitive gamete/embryo donation must be restricted [30, 34, 46–49, 52, 53]. In Canada and U.S.A oocyte donation is limited to six times during life and sperm donation is limited to 25 children per one million population and 25 pregnancies per 800,000 population, respectively [30, 46, 47, 53]. In the U.K and Australia, repetitive donation is limited based on the number of recipient families [34, 48, 49]. HFEA set the maximum number of family numbers that can be created using the same donor to 10 families [34].

Gamete and/or embryo donors might request to know about the outcome of their donations. It is recommended that fertility centers inform potential donors about if and how much information they can access about donation outcomes [43, 52]. Two guidelines suggest that donors have the right to know the number, sex, and age of the children resulting from their donation [34, 49].

Fertility centers must respect donors’ wishes in regard to set conditions for their donated gametes/embryos as long as these conditions are not against the non-discriminatory treatment guidelines, therefore based on some of the included guidelines donors can limit the number of families that will receive their gamete/embryo or they can donate to the recipient they know but they cannot limit their donation to a specific race, ethnicity or sexual orientation [34, 49, 51, 52].

Compensation and payment to gamete donors are controversial. While some guidelines prohibit compensation to donors [44, 45], the majority of guidelines consider compensation to donors to be fair and ethical [30, 34, 46, 47, 49, 51, 52, 54], but there are variations in the amount and mode of payment. HFEA’s ‘code of practice’ suggests a fixed amount of payment per donation for gamete donors, and also recommends that any expense resulting from donation side effects should be covered. Donors may receive payment in the form of benefits in kind, such as accessing treatments in the same fertility clinic [34]. ASRM guidelines recommend the compensation be a fair and specified amount, which would not interfere with the donor’s informed decision-making process. Three guidelines suggest that any cost related to donation’s side effects must be covered too [30, 52, 54]. Other guidelines suggest reimbursement for time and expenses [46, 47, 49, 51].

In addition to the ethical recommendations that were discussed, based on two guidelines provided by ASRM including Psychological guidelines for embryo donation and Guidance regarding gamete and embryo donation, staff members of a fertility center are prohibited from donating their gamete/embryo to that center in order to prevent conflict of interests [30, 44]. Also, some guidelines recommend if a person is considered ineligible for donation, that person have the right to know the reason, and centers should provide them with an explanation, counseling, and if necessary, referral for treatment [34, 44].

**Table 5:** Ethical considerations suggested by different guidelines for management and care of gamete/embryo donors

*	Name	Informed consent	Age limit	Repetitive donation restriction	Right to know the outcome	Conditional donation	Right to compensation
1	Guidelines for counselling in infertility [43]	+	-	-	+	-	-
2	Psychological guidelines for embryo donation [44]	+	+	-	-	-	No
3	Assisted Human Reproduction Counselling Practice Guidelines [45]	+	-	-	-	-	No
4	Guidelines for the Donation of Gametes and Embryos, Surrogacy and Preimplantation Genetic Diagnosis [46]	+	+	+	-	-	Reimbursement
5	Guidelines for Third Party Reproduction [47]	+	+	+	-	-	Reimbursement
6	Interests, obligations, and rights in gamete and embryo donation: an Ethics Committee opinion [52]	+	-	+	+	+	Yes
7	UK guidelines for the medical and laboratory procurement and use of sperm, oocyte and embryo donors [48]	+	+	+	-	-	-
8	Repetitive oocyte donation: a committee opinion [53]	-	-	+	-	-	-
9	Financial compensation of oocyte donors: an Ethics Committee opinion [54]	-	-	-	-	-	Yes
10	Guidance regarding gamete and embryo donation [30]	+	+	+	-	-	Yes
11	Code of practice (9th edition) [34]	+	+	+	+	+	Reimbursement
12	Code of practice for assisted reproductive technology units [50]	+	-	-	-	-	-
13	Good practice recommendations for information provision for those involved in reproductive donation [51]	+	-	-	-	+	Reimbursement
14	Ethical guidelines on the use of assisted reproductive technology [49]	+	+	+	+	+	Reimbursement

(‘+’ means that there are recommendations in the guideline about this topic, ‘- ‘means that this topic has not been mentioned in the guideline)

## Discussion

In this systematic review, we attempted to provide a summary of recommendations of assisted and third party reproduction guidelines in regards to management and care of gamete and embryo donors. Management and care provided for gamete and embryo donors were classified into four categories including screening, counseling, information provision, and ethical considerations.

It is important to note that relatively limited research has been undertaken to understand the gamete and embryo donors’ needs, expectations and experiences regarding care provided to them [26, 36, 55].

Genetic screening of potential donors is done in order to exclude donors, who are not considered eligible, but it is important to consider donors’ rights and needs. Based on the limited current research on donors’ experience regarding genetic screening, although donors understand and accept the necessity of genetic screening, they have some concerns about different aspects of screening such as its effect on their privacy, implications of the result on their life, ethics of genetic selectivity and ethics and implications of expanded genetic screening. Since genetic screenings do not benefit donors, there should be a balance between the burden of screening on donors, and its benefits for recipients and donor-conceived child [56–58].

Although guidelines have emphasized on the importance of counseling [30, 34, 43–45, 47, 49, 51], studies on donors’ experiences shows donors’ unmet needs for receiving proper counseling and/or support [13, 59, 60]. Results of the previous studies showed that the psychosocial needs of gamete/embryo donors for counseling and follow-up care are neglected in clinical settings [13, 59–61]. This is also the case of post-donation counseling. Although four guidelines recommend post-donation counseling [34, 43, 45, 51] and one guideline recommends follow-up care for oocyte donors [47], findings of various studies suggest that donors are not satisfied with the post donation counseling, follow-up, and care provided by the fertility clinics [17, 29, 36, 59, 61]. Donors need post-donation counseling regarding their experiences, disclosing donation to family members, receiving information about donor-conceived child, and/or contact with donor-conceived child, but some reports suggest that some donors do not receive the counseling or are not aware that post-donation counseling is available [32, 36, 62].

Also as mentioned before, in contrast to earlier guidelines about counseling of potential donors, which mainly focused on mental health screening for eligible donors, the latest guidelines separate counseling from mental screening and/or information provision [34, 48, 49, 51]. However, based on donors’ experiences, counseling in clinical settings is still used as a screening tool to determine their eligibility; therefore potential donors may not feel safe to discuss their special worries or problems [32, 63]; although it is possible for donors to seek counseling outside fertility clinics in order to manage their concerns or problems regarding donation. Counselors outside fertility clinics may have limited practical knowledge about donation and its implications on parties involved, but they cannot interfere with or stop the donation process even if they consider the potential donor ineligible for donation [64, 65]. So, it is important to understand the influence of counseling sessions outside fertility clinics paid by donors, on the donation process.

Also, donors must be counseled regarding the impact of donation and contact with donor-convinced child on their family; even though guidelines mentioned the importance of this topic, there is no specific guidance on how and when donors, and/or their family members should be counseled [32, 35, 66]. There are important topics regarding donation implications that must be discussed with donors prior to donation. Counselors not only should inform donors about possible future contact with the donor-conceived child/children, they should also make sure that donors understand the reasons behind the desire of donation offspring to contact them [67].

They also should receive counseling about the meaning of family and who is considered family member in the context of reproductive donation, since the understanding of parties involved in reproductive donation about family, and familial relations can be differ [68–70].

Additionally, donors should be counseled about their desired number of offspring, and for those who have donated multiple times, so there should be counseling on how to manage an overwhelming number of offspring [71, 72]. It is also important to notice that the research in this area is limited, therefore there is a need for further research on the meaning of family and its implication on the life of donors, recipients, and donor-conceived children. So, the necessity for developing needs-based guidelines regarding counseling of gamete/embryo donors is felt.

Another neglected but important aspect of both counseling and information provision is the topic of availability of commercial DNA testing, which can jeopardize donors’ anonymity. Donor-conceived children could be linked with donors or other genetically related family members of donors including their children through ancestry/DNA databases or some organizations. This can affect donor, donor’s family, donor-conceived child and the recipient family, significantly. Therefore, it is important to have clear guidelines about informing donors in the initial steps of donation about the unintentional disclosure of donation, and providing them with support about the management of this kind of disclosure within their own family and with the donor-convinced child; if and when it happens [32, 35, 73–75].

According to the reviewed guidelines, fertility centers are obligated to inform potential donors of all possible side effects related to gamete/embryo, however, the fact is that due to the lack of evidence, long-term side effects of donation are not completely identified [30, 34, 43–47, 50–52]. Studies on donors’ experiences and awareness showed that although most donors receive enough information about the short-term side effects of their donation, they still need more information regarding psychosocial side effects and potential physical/psychosocial long-term risks of donation [4, 13, 15, 59, 61, 76]. These studies also pointed to the need for longitudinal studies on donation side effects, in order to improve guidelines, recommendations, and informed consents [13, 15, 59, 76]. Also, informed consent forms must be transparent regarding the limited knowledge about the long-term side effects of donation especially among oocyte donors [77].

In relation to the ethico-legal aspects of donation, most of the guidelines have pointed out that donors have the responsibility to update their health status and contact information [30, 34, 45, 46, 49–52], but evidence shows that fertility clinic staffs are not fully aware of how these updating process should be conducted [78], therefore most gamete donors are rarely contacted to update their medical status. In cases that donors’ medical status is updated, there is also little to no guidance on who and how should inform the recipient parents [78]. Lack of accurate and comprehensive registry system, which includes and connects data of donors, recipients and all children born from a single donor together with the lack of binding regulations in this context, worsen the situation [79, 80].

Regarding donors’ rights, two guidelines recommended that donors must access information about donation offspring [34, 49], this is in line with the results of different studies which indicated that donors wish to be informed about the outcome of their donation [6, 59, 81].

One of the most controversial ethical aspects of gamete/embryo donation is compensation or payment to gamete donors. Most current guidelines consider compensation (not payment) to donors to be fair and ethical (28,29,31–33,35,36,38), but in clinical settings for example in the United States the amount of compensation can be negotiated, turning the donation process into a marketing for human gametes [82]. Those in favor of compensation with no fixated amount argue that it is a necessary step to ensure adequate supply for the growing demand of gamete donation [51, 54, 82, 83]. However, those against the payment (or compensation without limit), believe that payment in exchange for one’s gamete is a form of objectifying and commodifying humans, and increasing the cost of treatment causes inequality in access to treatment, so that is morally inappropriate [51, 82, 84, 85].

Another important ethico-legal issues that has been emphasized in the guidelines, is setting a limit on the optimal number of offspring per gamete donor [30, 34, 46–49, 52, 53]. But guidelines, especially in places where there is no registry system and anonymous donation is still an option, like U.S.A, are not clear on how they will keep track of the number of offspring born from a donor [79]. Also, since there is a possibility of cross-border donation, the limitation of offspring numbers should include overseas born children, also there should be guidance regarding how the number of live births resulted from donations of a single donor will be traced. Donors’ wishes regarding the number of children resulting from their donation should also be considered, since based on previous research most donors rather are keen to limit the number of offspring to a lower number like 10 [71, 72]. Also, based on the lack of accurate registry system, it will not be possible for donors to access information about all offspring resulted from their donation. Therefore, painting a realistic picture of donation practices should be at the core of setting guidelines and recommendations so that they are based on reality.

Also, as aforementioned, it should be noted legally that most guidelines are non-bindings [20]. This raises an issue when fertility centers do not follow the noted guidelines. As pervious research demonstrate, in the cases of non-binding guidelines and/or regulations, some fertility centers may not completely comply with the guidelines [3, 86, 87]. So it seems necessary to launch regulatory bodies to encourage awareness and use of the guidelines and to monitor implementing guidelines into practice to assist practitioners and patients’ decisions regarding appropriate healthcare for specific clinical issues [88].

The strength of this study is that to the best of our knowledge, this is the first study to review current assisted and third-party reproduction guidelines regarding management and care of donors. A limitation of this study was that only documents written in English were searched and included in the study. Additionally, although it was endeavored to adopt a comprehensive and systematic search strategy to find as many relevant documents as possible, there is still a possibility of missing data due to limited search in gray literature, including materials produced by organizations outside of the traditional commercial or academic publishing such as some of organizations affiliated with the International Federation of Fertility Societies.

## Conclusion

This review identified that management and care provided by assisted and third party reproduction guidelines for gamete and embryo donors were classified into four categories including screening, counseling, information provision, and ethical considerations. Nevertheless, there is a gap between guidelines and clinical practice regarding management and care of gamete and embryo donors. In order to inform current practice by developing evidence-based guidelines, well-designed research must be carried out to fill the knowledge gap about gamete and embryo donors’ needs, psychosocial effects of donation, long-term effects of donation on donors, donors' follow-up care, and ethical aspects of donation.

### Supplementary Information

Supplementary Material 1.

Supplementary Material 2.

## Data Availability

Data is provided within the manuscript or supplementary information files.

## References

[1] American Society for Reproductive Medicine. Gamete (Eggs And Sperm) And Embryo Donation. 2014. https://www.reproductivefacts.org/news-and-publications/patient-fact-sheets-and-booklets/documents/fact-sheets-and-info-booklets/gamete-eggs-and-sperm-and-embryo-donation/. Cited 2021 Oct 7.

[2] European Society of Human Reproduction and Embryology (ESHRE). Information provision in donation, Good practice recommendations for information provision for those using and participating in reproductive donation, Guidelines under development. 2021. https://www.eshre.eu/Guidelines-and-Legal/Guidelines/Guidelines-in-development/Information-provision-in-donation. Cited 2022 Jan 7.

[3] Richards M, Pennings G, Appleby JB. Reproductive Donation: Practice, Policy and Bioethics. First edition. Cambridge: Cambridge University Press; 2012.

[4] Tulay P, Atılan O (2019). Oocyte donors’ awareness on donation procedure and risks: A call for developing guidelines for health tourism in oocyte donation programmes. J Turkish German Gynecol Assoc.

[5] Nordqvist P (2019). Un/familiar connections: on the relevance of a sociology of personal life for exploring egg and sperm donation. Sociol Health Ill.

[6] Borgstrøm MB, Nygaard SS, Danielsen AK, Kesmodel US (2019). Exploring motivations, attitudes and experiences of oocyte donors: a qualitative study. Acta Obstet Gynecol Scand.

[7] Gilman L (2018). Toxic money or paid altruism: the meaning of payments for identity-release gamete donors. Sociol Health Ill.

[8] Adib Moghaddam E, Kazemi A, Kheirabadi G, Ahmadi SM (2020). Self-image and social-image of the donors: Two different views from oocyte donors’ eyes. J Health Psychol.

[9] Oppenheimer D, Oppenheimer A, Vilhena S, Von Atzingen A (2018). Shared Oocyte Donation: Ideas and Expectations in a Bioethical Context Based on a Qualitative Survey of Brazilian Women. Revista Brasileira de Ginecologia e Obstetrícia / RBGO Gynecology and Obstetrics.

[10] Nachtigall RD, Mac Dougall K, Harrington J, Duff J, Lee M, Becker G (2009). How couples who have undergone IVF decide what to do with surplus frozen embryos.. Fertility and sterility.

[11] Latifnejad Roudsari R, Hadizadeh Talasaz F, Simbar M, Khadem Ghaebi N (2014). Challenges of Donor Selection: The Experiences of Iranian Infertile Couples Undergoing Assisted Reproductive Donation Procedures.. The Iranian Journal of Obstetrics, Gynecology and Infertility.

[12] Ghorbani F, Latifnejad Roudsari R (2022). A Narrative Review of the Legal, Jurisprudential and Ethical aspects of Embryo Donation: Implications for Infertility Counselling. J Midwifery Reproductive Health.

[13] Adib Moghaddam E, Kazemi A, Kheirabadi G, Ahmadi SM (2021). Psychosocial consequences of oocyte donation in donors: a systematic review. Eur J Obstet Gynecol Reproductive Biology.

[14] Gonzalo J, Perul M, Corral M, Caballero M, Conti C, García D (2019). A follow-up study of the long-term satisfaction, reproductive experiences, and self-reported health status of oocyte donors in Spain. Eur J Contracept Reprod Health Care.

[15] Bracewell-Milnes T, Saso S, Bora S, Ismail AM, Al-Memar M, Hamed AH (2016). Investigating psychosocial attitudes, motivations and experiences of oocyte donors, recipients and egg sharers: a systematic review. Human reproduction update.

[16] Samorinha C, de Freitas C, Silva S. Donor-centred care: the facilitating and constraining factors experienced by gamete donors in a public bank. Human Fertility. 2021 10.1080/14647273.2021.1962987. Cited 2021 Oct 29. 10.1080/14647273.2021.1962987 34355619

[17] Hogan RG, Hammarberg K, Wang AY, Sullivan EA. ‘Battery hens’ or ‘nuggets of gold’: a qualitative study on the barriers and enablers for altruistic egg donation. Human Fertility. 2021. https://pubmed.ncbi.nlm.nih.gov/33451270/. Cited 2021 Jul 21 . 10.1080/14647273.2021.1873430 33451270

[18] Klein WW (2002). Current and future relevance of guidelines. Heart.

[19] Kish MA (2001). Guide to Development of Practice Guidelines. Clinical Infectious Diseases.

[20] Institute for Quality and Efficiency in Health Care (IQWiG). What are clinical practice guidelines?. Cologne, Germany. Institute for Quality and Efficiency in Health Care (IQWiG); 2016. https://www.ncbi.nlm.nih.gov/books/NBK390308/. Cited 2023 Jul 8.

[21] Wangler J, Jansky M (2021). What is the significance of guidelines in the primary care setting? Results of an exploratory online survey of general practitioners in Germany.. Wiener Medizinische Wochenschrift.

[22] Andersen BL, Dorfman CS (2016). Evidence-based psychosocial treatment in the community: considerations for dissemination and implementation. Psycho-Oncology.

[23] Mertz M, Strech D (2014). Systematic and transparent inclusion of ethical issues and recommendations in clinical practice guidelines: a six-step approach. Implementation Science.

[24] World Health Organization. WHO handbook for guideline development. Geneva: World Health Organization; 2012. https://apps.who.int/iris/bitstream/handle/10665/75146/9789241548441_eng.pdf. Cited 2024 Mar 21.

[25] Skoog Svanberg A, Lampic C, Gejerwall A, Gudmundsson J, Karlström P, Solensten N (2013). Gamete donors’ satisfaction; gender differences and similarities among oocyte and sperm donors in a national sample. Acta Obstet Gynecol Scan.

[26] Samorinha C, de Freitas C, Silva S (2023). Donor-centred care: the facilitating and constraining factors experienced by gamete donors in a public bank. Human Fertility.

[27] Newton CR, McDermid A, Tekpetey F, Tummon IS (2003). Embryo donation: attitudes toward donation procedures and factors predicting willingness to donate*. Human Reproduction.

[28] Goedeke S, Daniels K, Thorpe M, Du Preez E (2015). Building extended families through embryo donation: the experiences of donors and recipients. Hum Reprod.

[29] Williams RA, Machin LL (2018). Rethinking gamete donor care: A satisfaction survey of egg and sperm donors in the UK. PLOS ONE.

[30] American Society for Reproductive Medicine, Practice Committee of the American Society for Reproductive Medicine and the Practice Committee for the Society for Assisted Reproductive Technology. Guidance regarding gamete and embryo donation. Fertility and Sterility. 2021;115:1395–410. https://linkinghub.elsevier.com/retrieve/pii/S0015028221000789. Cited 2022 Jan 7.

[31] Blyth E (2012). Guidelines for infertility counselling in different countries: Is there an emerging trend?. Human Reproduction.

[32] Donation EWG, Kirkman-Brown R, Calhaz-Jorge J, Dancet C, Lundin EAF, Martins K. Good practice recommendations for information provision for those involved in reproductive donation†. Human Reproduction Open. 2022;2022 10.1093/hropen/hoac001. Cited 2023 Jul 20. PMC8847071 35178481

[33] European Society of Human Reproduction and Embryology (ESHRE). Guidelines for Counselling in Infertility. 2001. https://www.eshre.eu/Specialty-groups/Special-Interest-Groups/Psychology-Counselling/Archive/Guidelines.aspx. Cited 2022 Jan 7.

[34] Human Fertilisation and Embryology Authority. The Code of Practice (HFEA) 9th Edition. 2021. https://portal.hfea.gov.uk/knowledge-base/read-the-code-of-practice/. Cited 2023 Jul 16

[35] Blyth E, Crawshaw M, Frith L, van den Akker O (2017). Gamete donors’ reasons for, and expectations and experiences of, registration with a voluntary donor linking register. Human Fertility.

[36] Loyal S, Hudson N, Culley L, Weis C (2023). The experience of counselling for UK egg providers. Counselling and Psychotherapy Research.

[37] Perler L, Schurr C (2021). Intimate Lives in the Global Bioeconomy: Reproductive Biographies of Mexican Egg Donors. Body & Society.

[38] Page MJ, McKenzie JE, Bossuyt PM, Boutron I, Hoffmann TC, Mulrow CD (2021). The PRISMA 2020 statement: An updated guideline for reporting systematic reviews. PLOS Medicine.

[39] Khan KS, Kunz R, Kleijnen J, Antes G (2003). Five Steps to Conducting a Systematic Review. J R Soc Med.

[40] Zeng X, Zhang Y, Kwong JSW, Zhang C, Li S, Sun F (2015). The methodological quality assessment tools for preclinical and clinical studies, systematic review and meta-analysis, and clinical practice guideline: a systematic review. J Evid Based Med.

[41] Brouwers MC, Kho ME, Browman GP, Burgers JS, Cluzeau F, Feder G (2010). AGREE II: advancing guideline development, reporting and evaluation in health care. CMAJ.

[42] Popay J, Roberts H, Sowden A, Petticrew M, Arai L, Rodgers M, Britten N, Roen KDS. Guidance on the conduct of narrative synthesis in systematic reviews. 2006. https://citeseerx.ist.psu.edu/document?repid=rep1&type=pdf&doi=ed8b23836338f6fdea0cc55e161b0fc5805f9e27. Cited 2024 Apr 20.

[43] Boivin J, Appleton TC, Baetens P, Baron J, Bitzer J, Corrigan E (2001). Guidelines for counselling in infertility: outline version. Hum Reprod.

[44] American Society for Reproductive Medicine (2004). Psychological guidelines for embryo donation. Fertility and Sterility.

[45] Canadian Fertility and Andrology Society, (CSIG) CSIG. Assisted Human Reproduction Counselling Practice Guidelines. 2009. https://cfas.ca/_Library/clinical_practice_guidelines/CSIG_Counselling_Practice_Guidelines_August_2009_.pdf.

[46] Commission de l’éthique de la science et de la technologie. ETHICS AND ASSISTED PROCREATION: Guidelines for the Donation of Gametes and Embryos, Surrogacy and Preimplantation Genetic Diagnosis [Internet]. Gouvernement du Quebec. 2009. p. 1–244. https://cfas.ca/_Library/clinical_practice_guidelines/Ethics_Assisted_Procreation_Guidelines_QUEGOVT.pdf. Cited 2023 Jul 18.

[47] Canadian Fertility and Andrology Society. Guidelines for Third Party Reproduction. 2016. https://cfas.ca/_Library/clinical_practice_guidelines/Third-Party-Procreation-AMENDED-.pdf. Cited 2023 Jul 20.

[48] Clarke H, Harrison S, Perez MJ, Kirkman-Brown J (2021). UK guidelines for the medical and laboratory procurement and use of sperm, oocyte and embryo donors (2019). Human Fertility.

[49] National Health and Medical Research Council. Ethical guidelines on the use of assisted reproductive technology. 2023. https://www.nhmrc.gov.au/about-us/publications/art. Cited 2023 Jul 20.

[50] The Reproductive Technology Accreditation Committee (RTAC). Codes of Practice. Fertility Society of Australia and New Zealand. 2021. https://www.fertilitysociety.com.au/rtac-australia-new-zealand/. Cited 2023 Jul 20.

[51] ESHRE Working Group on Reproductive Donation, Kirkman-Brown J, Calhaz-Jorge C, Dancet EA, Lundin K, Martins M, Tilleman K, Thorn P, Vermeulen N, Frith L. Good practice recommendations for information provision for those involved in reproductive donation. Hum Reprod Open. 2022;2022(1):1–26, hoac001. https://academic.oup.com/hropen/article/doi/10.1093/hropen/hoac001/6528996. 10.1093/hropen/hoac001 PMC8847071 35178481

[52] Daar J, Collins L, Davis J, Francis L, Gates E, Ginsburg E (2019). Interests, obligations, and rights in gamete and embryo donation: an Ethics Committee opinion. Fertility and Sterility.

[53] American Society for Reproductive Medicine. Practice Committee of the American Society for Reproductive Medicine and the Practice Committee for the Society for Assisted Reproductive Technology. Repetitive oocyte donation: a committee opinion. Fertil Steril. 2020;113:1150–3. https://linkinghub.elsevier.com/retrieve/pii/S001502822030306X. Cited 2023 Jul 18.

[54] American Society for Reproductive Medicine. The Ethics Committee of the American Society for Reproductive Medicine. Financial compensation of oocyte donors: an Ethics Committee opinion. Fertil Steril. 2021;116:319–25. https://linkinghub.elsevier.com/retrieve/pii/S0015028221002454. Cited 2023 Jul 20. 10.1016/j.fertnstert.2021.03.040 33910756

[55] Svanberg AS, Sydsj€ G, Lampic C (2020). Psychosocial aspects of identity-release gamete donation – perspectives of donors, recipients, and offspring. Upsala Journal of Medical Sciences.

[56] Pennings G (2020). Expanded carrier screening should not be mandatory for gamete donors. Human Reproduction.

[57] Amor DJ, Kerr A, Somanathan N, McEwen A, Tome M, Hodgson J (2018). Attitudes of sperm, egg and embryo donors and recipients towards genetic information and screening of donors. Reproductive Health.

[58] Karpin I, Mykitiuk R. Reimagining disability: the screening of donor gametes and embryos in IVF. J Law Biosci. 2021;8. https://academic.oup.com/jlb/article/doi/10.1093/jlb/lsaa067/5918885. Cited 2024 Mar 21. 10.1093/jlb/lsaa067 PMC8366717 34408898

[59] Visser M, Mochtar MH, De Melker AA, Van Der Veen F, Repping S, Gerrits T (2016). Psychosocial counselling of identifiable sperm donors. Human Reproduction.

[60] Blakemore JK, Voigt P, Schiffman MR, Lee S, Besser AG, Fino ME (2019). Experiences and psychological outcomes of the oocyte donor: a survey of donors post-donation from one center. J Assist Reprod Genet.

[61] Van den Broeck U, Vandermeeren M, Vanderschueren D, Enzlin P, Demyttenaere K, D’Hooghe T (2013). A systematic review of sperm donors: demographic characteristics, attitudes, motives and experiences of the process of sperm donation. Human Reproduct Update.

[62] Hammarberg K, Carmichael M, Tinney L, Mulder A (2008). Gamete donors’ and recipients’ evaluation of donor counselling: a prospective longitudinal cohort study.. Aust N Z J Obstet Gynaecol.

[63] Braverman AM (2015). Mental health counseling in third-party reproduction in the United States: Evaluation, psychoeducation, or ethical gatekeeping? Fertility and Sterility.

[64] Patel A, Sharma PVN, Kumar P (2018). Role of mental health practitioner in infertility clinics: A review on past, present and future directions. J Human Rep Sci.

[65] Benward J (2015). Mandatory counseling for gamete donation recipients: ethical dilemmas. Fertil Steril.

[66] Bolt S, Postema D, van der Heij A, Maas BM (2021). Anonymous Dutch sperm donors releasing their identity. Human Fertility.

[67] Indekeu A, Maas AJBM, McCormick E, Benward J, Scheib JE (2021). Factors associated with searching for people related through donor conception among donor-conceived people, parents, and donors: a systematic review. F&S Reviews.

[68] van den Akker O (2006). A review of family donor constructs: Current research and future directions. Human Reproduction Update.

[69] Halcomb L (2020). Who Counts as Family? Gamete donation and the construction of family forms in medical markets. J Family Issues.

[70] Kelly F, Dempsey D, Power J, Bourne K, Hammarberg K, Johnson L (2019). From Stranger to Family or Something in Between: Donor Linking in an Era of Retrospective Access to Anonymous Sperm Donor Records in Victoria, Australia. Int J Law, Policy Family.

[71] Sydsjö G, Lampic C, Bladh M, Svanberg AS (2014). Oocyte and sperm donors’ opinions on the acceptable number of offspring. Acta Obstet Gynecol Scand.

[72] Nelson MK, Hertz R, Kramer W (2016). Gamete donor anonymity and limits on numbers of offspring: the views of three stakeholders. J Law Biosci.

[73] McGovern PG, Schlaff WD (2018). Sperm donor anonymity: a concept rendered obsolete by modern technology. Fertility and Sterility.

[74] Klotz M (2016). Wayward Relations: Novel Searches of the Donor-Conceived for Genetic Kinship. Medical Anthropology.

[75] Harper JC, Kennett D, Reisel D (2016). The end of donor anonymity: how genetic testing is likely to drive anonymous gamete donation out of business. Human Reproduction.

[76] Tober D, Garibaldi C, Blair A, Baltzell K (2021). Alignment between expectations and experiences of egg donors: what does it mean to be informed?. Reproductive Biomedicine & Society Online.

[77] Schneider J, Lahl J, Kramer W (2017). Long-term breast cancer risk following ovarian stimulation in young egg donors: a call for follow-up, research and informed consent.. Reprod Biomed Online.

[78] Isley L, Callum P, HEALTHCARE PROVIDER PREFERENCES RELATED TO DISTRIBUTION OF GAMETE, DONOR MEDICAL HISTORY UPDATES (2020). Healthcare provider preferences related to distribution of gamete, donor medical history updates. Fertility and Sterility.

[79] Sabatello M, .S. (2015). Regulating Gamete Donation in the U: Ethical, Legal and Social Implications. Laws.

[80] Luetkemeyer L. Who’s Guarding the Henhouse and What Are They Doing with the Eggs (and Sperm)? A Call for Increased Regulation of Gamete Donation and Long-Term Tracking of Donor Gametes. Saint Louis University Journal of Health Law & Policy. 2010;3. https://scholarship.law.slu.edu/jhlp/vol3/iss2/8. Cited 2024 Apr 19.

[81] Graham S, Jadva V, Freeman T, Ahuja K, Golombok S (2016). Being an identity-release donor: a qualitative study exploring the motivations, experiences and future expectations of current UK egg donors.. Human Fertility.

[82] Tober D, Pavone V, Lafuente-Funes S, Konvalinka N, Eggonomics. Vitrification and bioeconomies of egg donation in the United States and Spain. Med Anthropol Quart. 2023. Available from: https://anthrosource.onlinelibrary.wiley.com/doi/10.1111/maq.12767. Cited 2024 Apr 20. 10.1111/maq.12767 37229598

[83] Shapiro DB (2018). Payment to egg donors is the best way to ensure supply meets demand. Best Pract Res Cli Obstet Gynaecol.

[84] Cornthwaite K, Goedeke S, Shepherd D, Rodino I. Student views on recognition and payment options for gamete donation in New Zealand. Australian and New Zealand J Obstet Gynaecol. 2023. https://obgyn.onlinelibrary.wiley.com/doi/10.1111/ajo.13702. Cited 2024 Apr 20. 10.1111/ajo.13702 37221091

[85] Goedeke S, Shepherd D, Rodino IS (2022). Fertility stakeholders’ concerns regarding payment for egg and sperm donation in New Zealand and Australia. Reproductive Biomed Soc Online.

[86] Alberta HB, Berry RM, Levine AD (2013). Compliance with donor age recommendations in oocyte donor recruitment advertisements in the USA. Reprod Biomed Online.

[87] Frith L, Blyth E (2014). Assisted reproductive technology in the USA: is more regulation needed?. Reprod Biomed Online.

[88] Pereira VC, Silva SN, Carvalho VKS, Zanghelini F, Barreto JOM (2022). Strategies for the implementation of clinical practice guidelines in public health: an overview of systematic reviews. Health Res Policy Syst..

