# Supplementary material for: A systematic review of assisted and third-party reproduction guidelines regarding management and care of donors
Source: Reprod Health. 2024 Jun 1;21:75. doi: 10.1186/s12978-024-01804-2 (PMC11143578; doi:10.1186/s12978-024-01804-2)
Supplement: Supplementary file 1 — Supplementary Material 1. [file 12978_2024_1804_MOESM1_ESM.pdf]

Additional file 1. Search strategy for databases

*	Database	Search strategy	Number of articles
1	Scopus	((TITLE-ABS-KEY ("gamete donation") OR TITLE-ABS-KEY ("embryo donation") OR TITLE-ABS-KEY ("third-party reproduction"))) AND ((TITLE-ABS-KEY ("committee opinion") OR TITLE-ABS-KEY ("best practice") OR TITLE-ABS-KEY (guideline)))	154
2	PubMed	("gamete donation"[All Fields] OR "embryo donation"[All Fields] OR "third-party reproduction"[All Fields]) AND ("guideline"[Publication Type] OR "guidelines as topic"[MeSH Terms] OR "guideline"[All Fields] OR "committee opinion"[All Fields] OR "best practice"[All Fields])	59
3	Web of science	"gamete donation" (All Fields) OR "embryo donation" (All Fields) OR "third party reproduction" (All Fields)) AND "committee opinion" (All Fields) OR "best practice" (All Fields) OR guideline (All Fields)	104
