# Supplementary material for: A systematic review of assisted and third-party reproduction guidelines regarding management and care of donors
Source: Reprod Health. 2024 Jun 1;21:75. doi: 10.1186/s12978-024-01804-2 (PMC11143578; doi:10.1186/s12978-024-01804-2)
Supplement: Supplementary file 2 — Supplementary Material 2. [file 12978_2024_1804_MOESM2_ESM.pdf]

Additional file 2. Quality assessment score of clinical and ethical guidelines regarding gamete and embryo donors

*	Name	Domains score in percent						Overall Quality score
		1	2	3	4	5	6	
1	Guidelines for counselling in infertility [43]	61	56	12.5	36	21	75	3 (poor)
2	Psychological guidelines for embryo donation [44]	39	28	12.5	28	0	83	2 (very poor)
3	Assisted Human Reproduction Counselling Practice Guidelines [45]	42	50	0	31	0	92	2 (very poor)
4	Guidelines for the Donation of Gametes and Embryos, Surrogacy and Preimplantation Genetic Diagnosis [46]	72	67	37.5	75	19	96	3 (poor)
5	Guidelines for Third Party Reproduction [47] *	75	78	44	61	31	100	5 (good)
6	Interests, obligations, and rights in gamete and embryo donation: an Ethics Committee opinion [52]	75	75	46	75	23	100	3 (poor)
7	UK guidelines for the medical and laboratory procurement and use of sperm, oocyte and embryo donors [48]	75	75	46	75	23	100	3 (poor)
8	Repetitive oocyte donation: a committee opinion [53]	81	67	14	53	8	100	3 (poor)
9	Financial compensation of oocyte donors: an Ethics Committee opinion [54]	86	64	7	69	0	100	3 (poor)
10	Guidance regarding gamete and embryo donation [30]	86	72	53	78	56	100	6 (very good)
11	Code of practice (9th edition) [34]	86	72	47	81	67	100	5 (good)
12	Code of practice for assisted reproductive technology units [50]	75	72	27	83	60	100	5 (good)
13	Good practice recommendations for information provision for those involved in reproductive donation [51]	100	92	62.5	89	69	100	6 (very good)
14	Ethical guidelines on the use of assisted reproductive technology [49]	81	72	33	78	31	100	5 (good)
